# Coronavirus infection and kidney disease: a review of current and emerging evidence

**DOI:** 10.11604/pamj.2020.37.149.23655

**Published:** 2020-10-13

**Authors:** Oghenekaro Godwin Egbi, Oluseyi Ademola Adejumo, Ayodeji Akinwumi Akinbodewa

**Affiliations:** 1 Nephrology Unit, Department of Internal Medicine, Niger Delta University Teaching Hospital, Okolobiri, Bayelsa State, Nigeria,; 2 Department of Internal Medicine, University of Medical Sciences, Ondo, Nigeria

**Keywords:** Acute kidney injury, coronavirus infection, COVID-19, kidney disease, Middle East respiratory syndrome, pandemic, severe acute respiratory syndrome

## Abstract

In November 2009, an outbreak of a new strain of coronavirus (later named severe acute respiratory syndrome coronavirus-2 (SARS-CoV-2) was first noticed in the city of Wuhan in China, rapidly spreading to assume pandemic proportions within a short period of time. The disease was subsequently designated as coronavirus disease-19 (COVID-19). The death toll has continued to rise with grave health and socio-economic implications for individuals, families and nations globally. Although the respiratory tract is primarily involved in this disease, kidney affectation is increasingly reported and has been shown to worsen the prognosis of the disease. Current evidence shows that kidney disease is not uncommon in patients with coronavirus infection especially in those with COVID-19 and may arise from a constellation of factors such as hypotension, sepsis, rhabdomyolysis, multi-organ failure, use of nephrotoxic medications as well as direct infection in some cases. Factors associated with acute kidney injury in coronavirus infected patients may include elderly age, male sex, presence of co-morbidities as well as pre-existing chronic kidney disease and end stage renal disease. Although, there is presently no effective treatment for COVID-19, there is room for conservative management, extracorporeal therapy and renal replacement therapy. The aim of this review was to integrate current and emerging evidences on renal disease resulting from COVID-19 and the previous epidemics of coronavirus infections including the Middle East Respiratory Syndrome (MERS) and the Severe Acute Respiratory Syndrome (SARS) caused by other strains of the virus.

## Introduction

Following a local outbreak of pneumonia of unknown cause that was first observed in the city of Wuhan, China around November 2019 and quickly determined to be caused by a novel coronavirus, later named severe acute respiratory syndrome coronavirus-2 (SARS-CoV-2), the infection has dramatically spread to virtually all the continents of the world, including Africa, and is currently provoking a severe global alarm. On 11^th^ March 2020, the World Health Organization declared the novel coronavirus disease (COVID-19) to be a global pandemic after more than 118,000 cases were reported in 114 countries with 4,291 deaths [1]. As at 24^th^ April (about six weeks later), the numbers had escalated to 2,686,785 cases and 184,681 deaths in 213 countries and territories worldwide [2]. Apart from the direct health effects of the pandemic, there are also socio-economic implications for nations and the world as a whole. Although the respiratory system seems to be the primary target of the coronavirus, renal failure does occur and is increasingly being identified as a risk factor for mortality in these patients [3]. There are however some uncertainties about the epidemiology, pathogenesis or clinical course of COVID-19. This review aims to provide a comprehensive update of evidence on the effects of coronavirus infections on the kidneys with emphasis on the novel coronavirus disease.

## Methods

This review was conducted using peered reviewed articles focusing on research on coronavirus infection and kidney disease. An electronic literature search was conducted in the following databases: Google, Google Scholar and PubMed. Key words that correspond to the thematic objectives of the review such as acute kidney injury, coronavirus infection, COVID-19, kidney disease, Middle East respiratory syndrome, pandemic, severe acute respiratory syndrome were used in the search. Eligible articles were included for review only when abstracts contained explicit information about the issues of interest. Full text of the relevant articles and literature were then accessed and read.

## Current status of knowledge

**Incidence of renal disease in coronavirus infection:** following the first major pandemic of the human coronavirus, a few studies have been carried out to determine the incidence of renal disease in infected patients. In a retrospective analysis of 536 SARS cases across several hospitals in Hong Kong, Chu *et al*. reported acute renal impairment in 6.7% of admitted patients occurring at a mean duration of 20 days [3]. Half of those with acute renal impairment had oliguria. Also, dipstick proteinuria was found in 84.6% of the 26 patients that developed acute renal failure though heavy proteinuria was uncommon. Contrary to what was initially thought, acute renal failure from SARS does not only result from rhabdomyolysis but from immunopathological damage as well [4]. Acute renal impairment in SARS is believed to be largely due to pre-renal or renal factors occurring on the background of multi-organ failure [3].

Unlike in the case of SARS, kidney disease in patients with MERS-CoV seems to result from direct kidney involvement by the virus. It is thought that the mechanism of renal injury may include kidney tropism [5]. The first confirmed MERS-CoV patient was reported to have died of progressive respiratory and renal failure [6]. Kidney impairment seems to be more incident in MERS-CoV than with SARS. In a retrospective analysis involving thirty MERS-CoV patients in Korea, Cha *et al*. reported acute kidney injury (AKI) in eight (26.7%) patients with a mean and median duration of 18 and 16 days respectively [7]. Albuminuria reported on two occasions occurred in 60% and 40% of respondents while dipstick hematuria was found in 22% and 19% of participants on two occasions. Fifteen (50.0%) patients showed a random urine albumin creatinine ratio (ACR) or protein creatinine ratio (PCR) more than 100 mg/g cr.

Recent reports indicate that, renal impairment is not uncommon in confirmed cases of the novel coronavirus infection [8] while initial reports from Wuhan, China suggested that the burden of AKI with COVID-19 was relatively low, ranging from 3% to 9% [9]. Subsequent studies demonstrated incidence rates as high as 15% [8]. Although patients with mild to moderate disease may be spared, a significant proportion of patients with severe COVID-19 may develop kidney abnormalities including proteinuria and hematuria. Li *et al*. reported that 63% of patients in their study had proteinuria while elevated values of serum creatinine and urea were present in 19% and 27% respectively [10]. This is not surprising as about half of these patients had severe disease. Similarly, Cheng *et al*. in an analysis of 710 consecutively hospitalized patients with COVID-19 in Wuhan, found out that 44% had proteinuria while 26.7% had hematuria on admission [11]. The prevalence of elevated serum creatinine, elevated blood urea nitrogen and estimated glomerular filtration rate < 60mls/min/1.73m^2^ were 15.4%, 14.1% and 13.1% respectively. During the study period, AKI occurred in 3.2%% of all patients with COVID-19. The authors concluded that kidney disease was highly prevalent in hospitalized patients with COVID-19, and emphasized the need for early detection and effective intervention.

**Nature of renal injury in coronavirus infections:** renal tissues of patients with SARS show predominantly acute tubular necrosis (ATN) [3, 12]. A post-mortem examination performed among seven patients who died from SARS revealed mild to severe ATN. Ding *et al*. reported focal necrosis of the kidneys and vasculitis of small veins in the renal interstitial tissue in their study of three autopsy specimens [13] while another study described hydropic degeneration and swelling of the tubules, protein casts, tubular fibrosis and atrophy as well as glomerular atrophy and compensatory hypertrophy in remnant glomeruli [14]. Glomerular changes were found in only one tissue and could have been due to other co-morbid conditions as most other studies have reported glomerular sparing in SARS. A few of these studies have also detected SARS RNA antibodies in the renal tubules and urine of infected patients [15, 16]. Prevalent conditions likely to induce ATN in SARS include hypotension (which may be refractory), bacterial sepsis, rhabdomyolysis, hypoxia, cytokine storm syndrome and multi-organ failure. Renal histopathological autopsy reports of patients who had MERS have revealed the presence of viral particles in renal tissues, especially in the proximal tubular epithelial cells [17]. However, these particles may not be seen in all cases [7, 18]. Cha *et al*. reported ATN as the main renal biopsy finding in a MERS-CoV survivor [7]. Other common features observed in MERS include acute tubulointerstitial nephritis and presence of proteinaceous and granular casts [7, 18]. It is also not usual to find evidence of glomerular pathology in these patients except where there are other co-existing medical conditions.

Recently, Su *et al*. carried out a postmortem histopathological analysis on 26 renal tissues of patients with severe COVID-19 who died from respiratory failure due to multiple organ dysfunction syndrome and reported diffuse proximal tubular injury with loss of brush border, vacuolar degeneration, and frank necrosis on light microscopy [19]. Other findings noted were prominent erythrocyte aggregation obstructing capillary lumen but there was no evidence of vasculitis, interstitial inflammation or hemorrhage. Electron microscopy showed clusters of coronavirus particles with distinctive spikes in tubular epithelium and podocytes. Also, there was up regulation of angiotensin converting enzyme-2 (ACE-2) and immuno-staining while SARS-CoV-2 nucleoprotein antibody was positive in tubules. The factors contributing to AKI in patients with COVID-19 therefore include direct virulence of the virus, systemic hypoxia, abnormal coagulation and possibly drug or hyperventilation-related rhabdomyolysis. Contrary to findings in patients with SARS and MERS, patients with severe COVID-19 may show evidence of glomerular pathology. According to Su *et al*. there are evidences that the novel coronavirus causes podocytopathy which may be responsible for proteinuria commonly seen in AKI patients with COVID-19 [19]. It is possible that kidney tropism of SARS-CoV-2 plays a central role in the pathogenesis of COVID-19.

**Figure 1:**
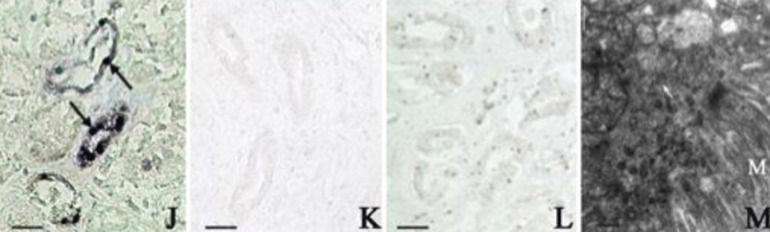
kidney tissue showing some abnormalities in SARS including; SARS genomic sequence in the tubular epithelium (arrows) of the distal tubules of the kidney of a patient who had SARS detected by in situ hybridization (J) compared with negative control in a SARS patient using an unrelated probe (K) and for a patient without SARS (L) where no positive signal is detected; EM image of the cytoplasm of a tubular epithelial cell in the kidney of a patient who had SARS showing clusters of SARS virus–like particles (arrows) in the cell (M)

**Figure 2:**
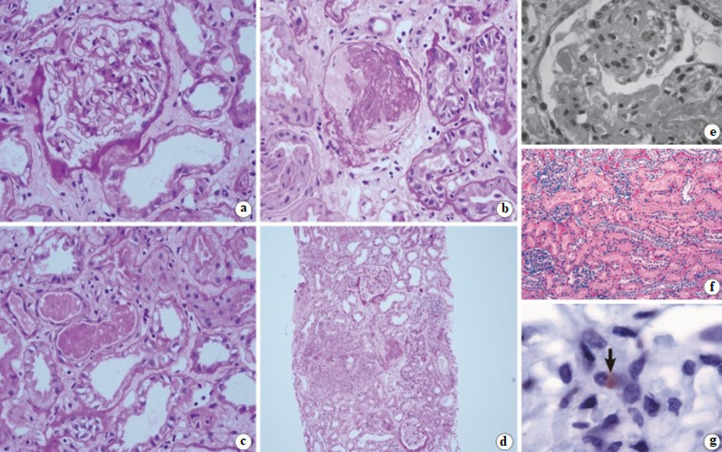
light microscopic findings of kidney tissues in MERS-CoV infection (H&E staining); a) glomerular pathology was not observed; b) acute tubular necrosis showing denuded tubular epithelial cells; c) some tubules showed proteinaceous cast formation; d) acute tubulointerstitial nephritis showing numerous inflammatory cells in tubules and the interstitium (a, b, c: magnification X 200, d: magnification X 40); e) proximal tubules with vacuolar degenerative alterations (magnification X 400) (17); f) moderate necrosis and detachment of tubular epithelium (magnification X 100) (18); g) viral antigen detected in the glomerular macrophage (arrow) (magnification X 1000)

**Figure 3:**
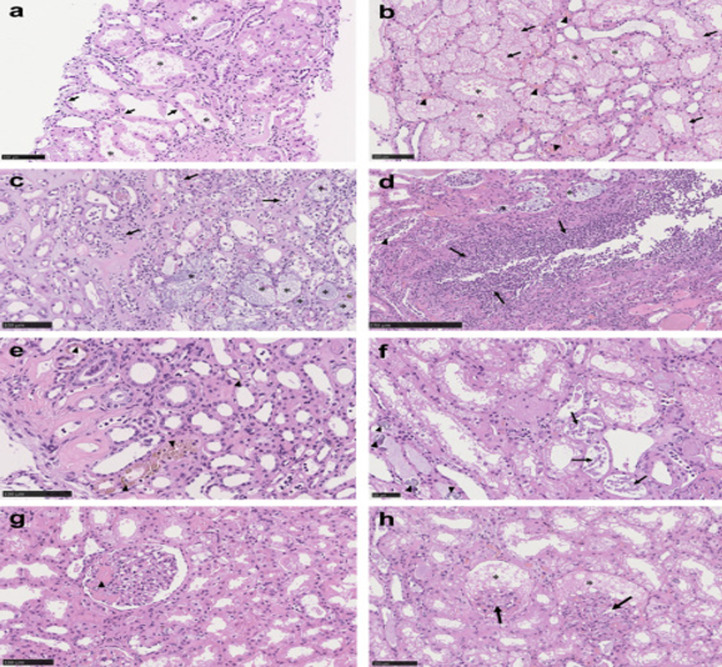
spectrum of pathologic abnormalities of kidneys from postmortems of patients with COVID-19; a, b) proximal tubules showed; a) loss of brush border and; b) vacuolar degeneration (arrows), with debris composed of necrotic epithelium in tubular lumens (asterisks); erythrocyte aggregates obstructing peritubular capillaries were frequently present (arrowheads); c, d) some cases showed infiltration of inflammatory cells in; c) tubules and; d) in 1 case, in an arcuate artery (arrows), with multiple foci of bacteria (asterisks) and white blood cell casts (arrowhead); e, f) occasional; e) hemosiderin granules and; f) deposits of calcium (arrowheads) were present in tubules with occasional pigmented casts (arrows); g, h) segmental fibrin thrombi were present in glomeruli (arrowhead), with ischemic glomerular contraction (arrows) with the accumulation of leaked plasma in Bowman´s space (asterisks); hematoxylin and eosin; Bars = (f) 50 μm; a-c, e, g, h) 100 μm, and d) 250 μm

**Figure 4:**
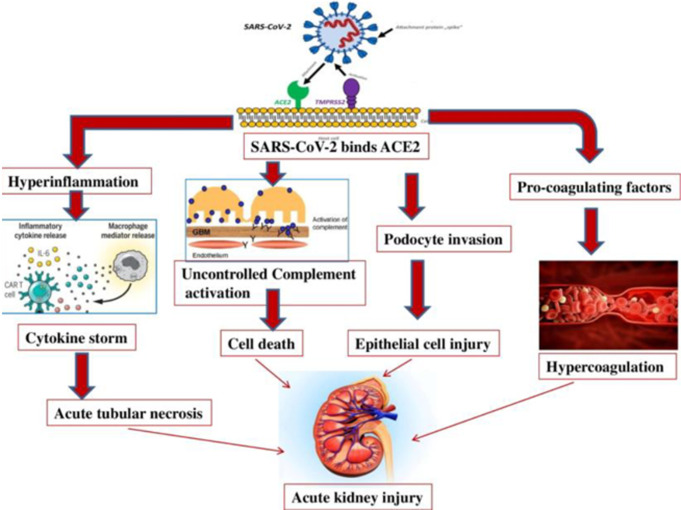
mechanism of acute kidney injury in COVID-19

**Mechanisms of renal injury:** the possible mechanisms that have been adduced for renal disorders in coronavirus infection include dehydration, sepsis leading to cytokine storm syndrome, rhabdomyolysis, and hypoxia [3]. Another mechanism observed to be at play is the direct cytopathic effect of the virus on tubular cells and particularly in the case of COVID-19, glomerular cells as well (Figure 1, Figure 2, Figure 3, Figure 4). Dehydration in these patients may be due to decreased fluid intake or fever especially in the elderly, and may result in reduction in renal perfusion and eventually acute tubular necrosis if the insult persists. Refractory hypotension, which frequently occurs in patients with multiple organ failure and bacterial sepsis especially in those on prolonged ventilatory support could predispose to AKI. Direct muscle invasion by SARS-CoV leading to rhabdomyolysis is a well-known mechanism for AKI in SARS. In the cytokine storm syndrome, elaboration of cytokines such as IL-6 and IL-8 induced by viral infections lead to adhesion of inflammatory cells to vascular endothelium, possibly leading to endothelium-dependent vasodilatation and subsequent renal injury [13, 20].

Dipeptidyl peptidase 4 (DPP 4) has been identified as the functional entry receptor for MERS-CoV [21]. Amongst the organs, the highest amount of DPP-4 activity per gram of tissue is found in the kidneys [22] though it is also highly expressed in the gut, liver and lungs [23]. In the kidneys, it is present in the brush border membrane, glomerular podocytes and capillaries. On the other hand, the SARS-CoV enters cells via the ACE-2 receptor which is also present in several cells in the body, including renal cells [24]. ACE-2 is an angiotensin converting enzyme homologue that negatively regulates the activated angiotensin system by degrading angiotensin II into angiotensin I-7, which normally exerts a protective effect on the kidneys, lungs and cardiovascular system [25]. It has also been recently shown that the novel virus, SARS-CoV-2 uses the same cell entry receptor with greater efficiency and binding affinity than does SARS-CoV [26]. In fact, it was discovered that the expression of these receptors in renal cells is several times more than in the pulmonary system [27]. This suggests that the renal cell may be more specifically targeted and infected by the coronavirus. In the case of COVID-19, it is also likely that renal injury may be induced through immune complex deposition in tubules and glomeruli or specific immunological changes brought about by the virus though virus-induced glomerulopathy does not seem to be a major pathway of the coronaviruses generally. Other mechanisms of renal failure in coronavirus infection may be the effect of pre-existing conditions or co-morbidities such as diabetes or hypertension which are common in these patients as well as use of nephrotoxic medications such as non-steroidal anti-inflammatory drugs.

**Factors associated with renal impairment in coronavirus infection:** factors shown to be significantly associated with acute renal impairment during the SARS epidemic were age, sex, and presence of acute respiratory distress syndrome, diabetes and heart failure [28]. The incidence of acute renal impairment was shown to be higher among the elderly [3, 28] and in the presence of co-morbidities such as diabetes, heart failure, respiratory failure, acute respiratory distress syndrome (ARDS) and multiple organ system failure [3, 29]. SARS patients who subsequently developed acute renal failure (ARF) also had lower plasma sodium and albumin with elevated alanine aminotransferase and plasma LDH on admission compared with those without ARF [3]. As in the case of SARS, older individuals are at a higher independent risk for the development of AKI in MERS and COVID-19 [7, 29, 30]. Other factors found to be associated with AKI in COVID-19 patients include male gender, multiple pre-existing comorbidities including hypertension, diabetes and cerebrovascular disease, greater severity of illness, increased infection indicators, lymphopenia, elevated D-dimer and impaired heart and liver functions [30].

**Effects of AKI on outcomes of patients with coronavirus infection:** the development of renal failure has been observed to be an important negative prognostic factor in SARS [3, 28]. AKI, together with diabetes and use of continuous renal replacement therapy were identified risk factors for mortality in the retrospective study among MERS-CoV patients [7]. Also, in another cohort of MERS-CoV patients, the presence of elevated creatinine > 1.5mg/dl and renal failure were associated with reduced survival of patients [29]. Elevated serum creatinine, elevated urea nitrogen, AKI, proteinuria and hematuria were independent risk factors for in-hospital death in COVID-19 after adjusting for age, sex, disease severity, leukocyte count and lymphocyte counts [11]. Renal disease is therefore considered an independent risk factor for COVID 19 patients´ in-hospital mortality [11, 30, 31].

**Dialysis therapy for AKI complicating coronavirus infection:** up to 5% of patients with COVID-19 will eventually require dialysis, usually from around the second week of infection [32]. This pattern is not particularly different from the previous MERS and SARS coronavirus epidemics. Various modalities of dialysis have been used for critically ill COVID-19 patients with mixed results. Peritoneal dialysis, hemofiltration and continuous renal replacement therapy (CRRT) have been used in different settings, depending largely on availability of equipment in such centres. Intermittent hemodialysis (IHD) may not be feasible in patients with hemodynamic instability. The indications for dialysis for AKI resulting from SARS infection are not different from those without SARS and may include uremia, electrolyte and acid-base imbalance as well as fluid retention [3]. A meta-analysis reported a lower mortality among patients with SARS who received CRRT compared with those placed on conventional therapy [33]. However, the indications for CRRT in these patients were severe sepsis / septic shock or ARDS, rather than renal failure. On the other hand, there is evidence that CRRT may be associated with increased mortality in MERS-CoV patients with severe sepsis and ARDS, though data is sparse [34]. Also, AKI and use of CRRT were identified as risk factors for mortality among patients with MERS-CoV [7]. It appears that CRRT does not confer any survival benefit in patients with MERS-CoV infection but rather may increase the odds of mortality. The reason for this is not clear and may warrant further studies.

CRRT remains preferred among critically ill COVID-19 patients with AKI especially in hemodynamically unstable patients. Even among patients who are hemodynamically stable and could tolerate IHD, CRRT or prolonged intermittent renal replacement therapy (PIRRT; also called sustained low-efficiency dialysis (SLED) should be performed if possible as it requires less support. CRRT has been shown to reduce all-cause mortality, even for critically ill patients with COVID including those on mechanical ventilation [35]. Apart from its effect in control of electrolytes and helping to regulate the acid base balance in the body, it is also believed to play a role in removal of inflammatory mediators and improvement of oxygen utilization in the fluid overloaded state. When facilities for HD or CRRT is unavailable, there may be need to consider treatment of AKI with peritoneal dialysis (PD). Patients with AKI who are treated with PD have similar rates of all-cause mortality, kidney function recovery, and infectious complications as patients treated with other modalities. PD usually requires less equipment and resources compared with other modalities. However, on the other hand, it may increase abdominal pressure, interfere with respiratory dynamics and worsen respiratory failure in patients who are on mechanical ventilation [36].

**Chronic kidney disease and coronavirus infection:** emerging evidences suggests that patients with pre-existing renal disease or chronic kidney disease (CKD) have an enhanced risk of severe COVID-19 infection [37]. In a meta-analysis involving 53,000 patients with COVID-19, CKD was associated with a 6 fold risk of severity of the infection when compared with those without CKD [38]. CKD patients should therefore take proper precautions to minimize exposure to the virus and should be closely monitored during this pandemic. Since the long-term effect of kidney injury on survivors is not yet known, patients who have recovered from AKI due to COVID-19 should be followed up for a period of time. Patients with proteinuria and / or haematuria in the absence of AKI should similarly be followed up. There are fears that the coronavirus pandemic could be followed by an epidemic of CKD and end stage renal disease (ESRD) [39].

**Patients on chronic dialysis and coronavirus infection:** during the SARS epidemic, it was observed that patients on maintenance dialysis had a higher risk of acquiring SARS compared with the general population [40]. They also presented with the same but milder clinical features with a delay in seeking medical attention. However, they tended to shed the virus for longer periods and had greater transfusion and longer hospitalization requirements. Mortality among them was however not significantly different from that of others [41]. Nosocomial outbreaks were a key feature of the MERS pandemic. During the 2015 MERS outbreak in Korea, 116 patients in three hemodialysis centres were incidentally exposed and subsequently isolated. The isolation methods used were shown to be effective in preventing secondary viral transmission [42]. The Korean Centre for Disease Control (KCDC), Korean Society of Nephrology and Korean Society of Dialysis Therapy subsequently developed a draft of MERS Clinical Practice Guidelines in 2015, which was amended in 2016 to prevent and control infection in hemodialysis units during nation-wide MERS-CoV epidemics [43]. It emphasized the need for hemodialysis for MERS-CoV patients to be done in a confined unit due to high risk of infection transmission among ESRD patients due to their low immunity.

Symptomatic COVID-19 patients should also be preferably dialyzed in a separate dialysis room [44]. If this is not available, the treatment should be provided at a location away from the main traffic flow. Care should be taken to ensure the patient is separated at least 2m from other patients in all directions [45]. Designated health care workers should be assigned to the patients while observing standard contact and droplet precautions, including isolation gowns, gloves, masks, and eye protection (with shields or goggles). Cough and sneeze etiquette should be ensured for symptomatic patients. Routine cleaning and disinfection of surfaces and instruments used as well as the dialysis machines and the entire dialysis station is also essential [45].

**Renal transplantation and coronavirus infection:** transplant recipients are uniquely predisposed to emerging infections. During the outbreak of SARS, transplant patients had more severe and rapidly progressive disease compared with the normal population [46]. Their higher viral burdens also suggested increased infectivity. Transplant programmes during epidemics of severe acute respiratory syndrome areas were also adversely affected because of donor concerns, recipient issues and resource problems [46]. Treatment of SARS in kidney transplant recipients is empirical. The usual immunosuppressant regimen might not need to be changed to prevent rejection; however, if additional immunosuppressant is to be used for SARS, there may be a need to reduce the dosages because of the possibility of overwhelming infections. Such patients should be isolated and those medical personnel taking care of them must be fully protected, with strict isolation measures enforced. Kidney transplant patients are more susceptible to SARS-CoV-2 infection and rapid progression of the disease compared to the general population [47]. This is because they are immunosuppressed by the medications they use and have other co-morbidities such as diabetes mellitus. The SARS-CoV-2 virus has been reported to reduce the population and functional capacity of T-cells which play pivotal roles in antiviral immunity [48]. These adverse effects on T-cells are more pronounced in transplant patients due to the effect of their immunosuppressant treatment [47]. It is therefore not surprising that these patients tend to have worse outcome. Mortality in transplant patients is higher than general population when they have COVID-19 infection [47]. There is need to institute aggressive treatment in them in order to have good outcome.

Also, there are growing concerns presently on the safety of new kidney transplantation in this period of COVID-19 pandemic. This is because newly transplanted patients are heavily immunosuppressed which may predispose them to severe and rapidly progressive SARS-CoV-2 infection with devastating consequences. In addition, the accuracy of the reverse transcription polymerase chain reaction which is the present gold standard of diagnosis of the infection may be affected by the method of sample collection, storage, transportation, processing and analysis [49, 50]. This may lead to transplantation of a patient with undetected SARS-CoV-2 infection in spite of routine screening. It is therefore advised that kidney transplantation be deferred until this pandemic is over. However, if there is a compelling indication to have a kidney transplant, the benefits and risks of the procedure to the patient may be weighed on individual basis by the transplant team before the decision is taken. A major strength of this review is the all-inclusive nature of the report. It sought to provide information on the epidemics of the human coronaviruses generally since they shared certain characteristics. It was not limited to a particular outbreak or strain of the virus even though more focus was on COVID-19. Most other reviews on the subject had been restricted to a particular outbreak of the coronavirus.

## Conclusion

Renal impairment occurs in patients with coronavirus infection, including the novel coronavirus disease-19 (COVID-19) which has become a global pandemic; as well as the previous outbreaks of severe acute respiratory syndrome (SARS) and the Middle East Respiratory Syndrome (MERS). Renal involvement may be due to a constellation of several factors such as sepsis, dehydration, multiple organ failure, nephrotoxic medications and direct effects of the virus and is usually associated with poor prognosis of the disease. Factors predisposing to acute kidney injury (AKI) in coronavirus-infected patients include older age, male sex and presence of co-morbidities. Other renal abnormalities such as proteinuria and haematuria are often seen especially in COVID-19. Patients with CKD, ESRD as well as renal transplant recipients are at higher risk of developing coronavirus infections compared with the general population. There is a need for early identification, prompt diagnosis, adequate management and follow up of kidney disease arising from this current outbreak.

### What is known about this topic

- SARS-CoV-2 infection is the newest novel coronavirus infection which has been declared a global pandemic by WHO and has devastating effects on most nations of the world;
- The coronavirus infection primarily affects the respiratory system; however the kidneys are also commonly affected;
- SARS-CoV-2 infection has overall negative effects on the course and outcomes of chronic kidney disease patients.

### What this study adds

- The study showed that renal disease is common in patients with coronavirus infections and is often associated with poor prognosis;
- The study showed that the clinical features of renal disease in patients with coronavirus infections have similar pattern irrespective of viral strain;
- The study identified risk factors for renal disease in patients with coronavirus virus. These are elderly age, male sex, presence of co-morbidities, preexisting CKD, being on maintenance dialysis and renal transplantation; the study also identified areas of future research to fill knowledge gaps in the mechanisms of renal disease in coronavirus-infected patients which may serve as a guide for therapy.
